# The polyphenol epigallocatechin gallate lowers circulating catecholamine concentrations and alters lipid metabolism during graded exercise in man: a randomized cross-over study

**DOI:** 10.1007/s00394-023-03092-1

**Published:** 2023-01-25

**Authors:** Rachel Churm, Liam M. Williams, Gareth Dunseath, Sarah L. Prior, Richard M. Bracken

**Affiliations:** 1 Applied Sports Technology Exercise and Medicine Research Centre (A-STEM), College of Engineering, Faculty of Science and Engineering, Swansea University, Engineering East, Bay Campus, Swansea, SA1 8EN UK; 2 Diabetes Research Group, Swansea University, Singleton Park, Swansea, UK; 3 Medical School, Swansea University, Grove Building, Swansea, UK

**Keywords:** Epigallocatechin gallate, Catecholamines, Substrate metabolism, Graded exercise

## Abstract

**Purpose:**

Physical exercise is shown to mitigate catecholamine metabolites; however, it is unknown if exercise-induced increases in sympatho-adrenal activity or catecholamine metabolites are influenced by ingestion of specific catechins found within green tea. This study explored the impact of epigallocatechin gallate (EGCG) ingestion on catecholamine metabolism during graded cycle exercise in humans.

**Methods:**

Eight males (22.4 ± 3.3 years, BMI:25.7 ± 2.4 kg.m^2^) performed a randomised, placebo-controlled, single-blind, cross-over trial after consumption (1450 mg) of either EGCG or placebo (PLAC) and performed graded cycling to volitional exhaustion. Venous bloods were taken at rest, 2 h post-ingestion and after every 3-min stage. Blood variables were analysed for catecholamines, catecholamine metanephrines and metabolic variables at rest, 2 h post-ingestion (POST-ING), peak rate of lipid oxidation (FATpeak), lactate threshold (LT) and peak rate of oxygen consumption (*V*O_2_peak). Data were analysed using SPSS (Version 26).

**Results:**

Resting catecholamine and metanephrines were similar between trials. Plasma adrenaline (AD) was lower in ECGC treatment group between trials at FATpeak (*P* < 0.05), LT (*P* < 0.001) and *V*O_2_peak (*P* < 0.01). Noradrenaline (NA) was lower under EGCG at POST (*P* < 0.05), FATpeak (*P* < 0.05), LT (*P* < 0.01) and *V*O_2_peak (*P* < 0.05) compared to PLAC. Metanephrines, glucose and lactate increased similarly with exercise intensity in both trials. Lipid oxidation rate was 32% lower in EGCG at FATpeak (EGCG 0.33 ± 0.14 vs. PLAC 0.49 ± 0.11 g.min^−1^, *P* < 0.05). Cycle time to exhaustion was similar (NS).

**Conclusion:**

Acute EGCG supplementation reduced circulating catecholamines but not; metanephrine, glucose or lactates, response to graded exercise. Lower circulating catecholamines may explain a lower lipid oxidation rate.

## Introduction

Catechins are polyphenolic flavonoids and are bioactive ingredients in green tea. The four main types of catechins found in green tea are epigallocatechin-3-gallate (EGCG), epicatechin-3-gallate (ECG), epigallocatechin (EGC) and epicatechin (EC) with the most abundant and pharmacologically active being EGCG [1]. Combined these catechins account for ~ 30% of dry weight of green tea leaves [2].

Green tea or its individual constituents have been shown to alter resting energy metabolism. Several short-term (< 3 days) supplementation studies have found an increase in resting energy expenditure [3–6]; however, this has not always been the case [7–9]. In studies that have found increased energy expenditure, this was related to an increase in lipid metabolism [4, 6]. For example, green tea ingestion (EGCG 245 mg.d^−1^, caffeine 270 mg.d^−1^) increased resting energy expenditure by 2.9% and lipid oxidation rates by 12% higher over the 24-h period compared with water [6]. Some studies have demonstrated no change in resting energy expenditure but found an increased lipid oxidation rate [7, 9]. However, altered lipid oxidation rates have not been replicated in all studies [3, 5]. Longer term interventional studies (6–12 weeks) have found greater body weight loss and/or prevention of weight gain after consuming green tea or green tea extract [10–12].

Whole body lipid oxidation rates may increase from resting values during submaximal exercise [13]. In this setting increased lipid combustion may allow for tissue glycogen sparing [14] and provide a greater calorific content per gram of fuel compared to carbohydrate. Acute green tea extract has been shown to alter lipid oxidation rates during exercise [15, 16]. The study by [16], examined how decaffeinated green tea extract (EGCG 366 mg) influenced metabolism during moderate intensity exercise. Over a 24-h period subjects consumed 3 capsules totalling 890 mg total polyphenols. Participants then performed a 30-min cycle at 60% *V*O2 max. The results of this study noted no change in energy expenditure but a 17% increase in lipid oxidation (0.35 ± 0.03 to 0.41 ± 0.03 g.min^−1^). Longer term supplementation studies have also noted increased lipid oxidation rates during exercise [17] however, most studies report no change in lipid utilisation rates following supplementation periods ranging from 1 to 28 days [18–23]. With increases in metabolic markers of fat oxidation under resting conditions following 7 days of ingestion of decaffeinated green tea extract (dGTE), is shown with an increase in 3-hydroxybutyrate at fasting and resting conditions following supplementation [21]. However, no increases in either glycerol and non-esterfied fatty acids were observed after 7 days of dGTE ingestion, indicating that dGTE did not enhance lipolysis. Furthermore, supplementation did not increase markers of fat oxidation during exercise. Interestingly, several studies have demonstrated efficacy of chronic green tea supplementation in losing weight [10–12]. However, [24] found no greater change in body mass, body composition, energy or substrate metabolism following supplementation when participants who were undergoing an energy restricted diet were also provided EGCG.

Few studies have explored the working mechanisms of green tea catechins in modulating resting or exercise energy metabolism that might translate to alterations in lipid metabolism or body mass loss. The sympatho-adrenal system is important in altering basal and exercise metabolism [25]. Circulating catecholamines can stimulate adipocyte and skeletal muscle hormone sensitive lipase to increase conversion of triglyceride to non-esterified fatty acids [25] and improve lipid combustion [26]. It has been hypothesized that the mechanism behind the short-term effects of green tea extract used in many studies may be directly related to the active green tea ingredient catechins. Catechins have been hypothesised to regulate the sympatho-adrenal system directly through inhibition of catechol-*O*-methyltransferase (COMT) activity [27] although this has been disputed [28]. COMT is an intracellular enzyme found in all tissues including skeletal muscle and adipose tissue, and degrades adrenaline and noradrenaline. Whilst in vitro results support flavonoid-mediated COMT inhibition [29] there is limited research evidence in vivo to support this potential mechanism. Although catecholamine metabolites like plasma metanephrine and normetanephrine have been shown to change in response to physical exercise [30] it is currently unknown if exercise-induced increases in sympatho-adrenal activity or catecholamine metabolites are influenced by ingestion of specific catechins found within green tea. Graded exercise is a useful modality to explore a well-characterised rise in energy metabolism and an increase in sympatho-adrenal activity [31].

Thus, the aim of this study was to explore the impact of acute ingestion of the polyphenol epigallocatechin gallate (EGCG) on catecholamine, catecholamine metabolite, systemic metabolic and cardio-vascular variables across a range of exercise intensities during graded cycle exercise in man.

## Methodology

### Participants

This study was conducted according to Declaration of Helsinki and all procedures involving human subjects were approved by the University Research Ethics Committee; PG/2014/28. Eight apparently healthy male participants (age: 22.3 ± 3.3 years, estimated body fat 15 ± 5.2%, BMI 25.7 ± 2.4 kg m^−2^) were recruited to this study. Participants were included if they had a habitual caffeine intake ≤ 400 mg.d^−1^ (less than four cups of tea/coffee or caffeinated soda beverages per day) and performed habitual exercise three to five times per week for 30–90 min per exercise session. Written informed consent was obtained from all participants and all participants completed a health screening questionnaire to ascertain their health status and to determine their eligibility to partake in this study accordance with American Heart Association/American College of Sports Medicine (AHA/ACSM) (clinicaltrials.gov NCT03199430, http://clinicaltrials.gov/show/NCT03199430).

### Experimental design

In a randomised, placebo-controlled, single-blind, cross-over design study participants completed two trials after acute consumption of either EGCG or a placebo (PLAC) supplement after an overnight fast. Principal investigator was responsible for generating the random allocation sequence, enrolling participants, and assigning interventions. Following a two-hour monitoring period participants performed a continuous graded cycle exercise test to volitional exhaustion. There was at least a 7-day washout period between trials. No reported losses and exclusions after randomisation in any group. Due to the non-clinical exploratory nature of the randomised cross-over study it was registered following ethical approval.

### Supplementation

Participants were randomly assigned to either the intervention or placebo trial. After overnight fast participants arrived to the laboratory and were observed ingesting two capsules each of EGCG (minimum 94% EGCG < 0.1% caffeine) from a commercially available brand (TEAVIGO™; TAIYO GmbH, 1450 mg) or a placebo (1450 mg Corn Flour). Capsules were weighed and sorted to within ± 5%. The supplement was consumed in two size 00 vegetarian gelatin capsules alongside a standardized amount of distilled water (200 ml).

### Experimental protocol

Prior to participation in the experimental trials participants were familiarized with the laboratory equipment and the test procedures. On the morning of the test participants reported to the Exercise Physiology Laboratory following an 8–10 h fast where measures of body mass (weighting scales; Seca 770 Digital Scales, Seca Ltd, Birmingham, UK), height (stadiometer; Holtain Stadiometer, Holtain Ltd, Cymrych, Wales) and estimated fat percentage using bioelectrical impedance analysis (Bodystat Quadscan 4000, Bodystat Ltd, Isle of Man, UK) were made whilst wearing minimal clothing.

Participants were then seated for a 10-min period while a cannula was inserted into an antecubital vein. This was connected to a three-way stopcock for the repeated collection of venous blood at rest and during the exercise test. Saline (2–3 ml) was infused regularly keep the cannula patent. After a 10-min rest period, a venous blood sample (7 ml) was collected into a lithium-heparinised vacutainer. In addition, baseline measures of heart rate (Polar RS800CX), and a 10-min sample of expired air (Jaeger Vyuntus CPX, Erich Jaeger GmbH, CareFusion Hoechbegh, Germany) were also taken. Expired air was collected throughout the duration of the exercise test and measured for volume, the fractional concentration of oxygen (F_E_O_2_%) and carbon dioxide (F_E_CO_2_%) (SentrySuite Software, Erich Jaeger GmbH, CareFusion Hoechberg, Germany). This allowed for the determination of volumes of O_2_ utilization and CO_2_ production. These data were then used to determine oxidative energy expenditure using principles of indirect calorimetry [32].

Following collection of resting parameters participants were remained in a semi-reclined position for two hours post ingestion of green tea extract. Then after a 5-min transition period, participants mounted a cycle ergometer (Lode Excalibur Sport Ergometer, Lode BV Groningen, The Netherlands) to perform a graded exercise test. Participants were instructed to cycle between 60 and 70 rpm at an initial power output of 60 Watts (W) with an increase in 30 W every 3 min. Verbal encouragement was provided to the participant throughout. Heart rate was measured constantly throughout the exercise test (Polar RS800CX) alongside respiratory gas measurements. Two and a half minutes into each 3-min stage, a rating of perceived exertion (RPE [33]) was taken from the participant and a venous blood sample obtained. The test continued until volitional exhaustion defined by the following criteria (1) cadence dropping below 50 rpm, (2) heart rate within 10 beats of age-predicted maximum, (3) levelling of VO_2_ though workload had increased. At this point cardio-respiratory variables were recorded and a final blood sample was taken at exhaustion. The participants dismounted the ergometer, reclined on a couch and were provided with water ad libitum. The indwelling cannula was removed and participants were monitored for 30 min before leaving the laboratory. No harms or unintended effects were reported in either group.

### Diet control

Participants were given food and physical activity diaries to complete in the 72 h prior to the first experimental trial and were strongly encouraged and reminded via SMS to replicate this diary prior to the second trial. Participants were also instructed to avoid alcohol, foods with high polyphenol content (i.e. fruits, dark chocolate and cereal bran), caffeinated beverages during this period. Participants were also instructed not to perform any physical activity in the 24 h period prior to each trial. The food diary was analysed using CompEat software (CompEat 5.7 Pro). In the 72 h preceding the first arm the macronutrient composition of the participants’ diets was carbohydrate 40.20%, fat 37.61% and protein 22.20%, this was replicated for the alternate arm of the study and included a 5% margin of error for diet replications across trial arms.

### Blood analyses

Venous blood samples were analysed immediately for lactate and glucose levels (Biosen C-Line, EKF Diagnostics). Thereafter, the remainder of the sample was centrifuged (Heraeus Megafuge 8, Thermo Scientific) for 10 min at 3,000 rpm with ~ 3 ml of plasma extracted into individual 1 ml microcentrifuge tubes and frozen immediately (− 80 °C) for later analysis of metanephrine, normetanephrine and catecholamine (adrenaline and noradrenaline) concentrations using commercially available enzyme linked absorbent assays (ELISA, Eagle Biosciences Inc, Nashua, New Hampshire, USA). The lower limit of detection for the adrenaline and noradrenaline assay was 5 pg ml^−1^ and 16 pg ml^−1^, respectively. Average intra-assay coefficients of variation were 8.35% for adrenaline and 9.7% for noradrenaline. Average recoveries of 97% and 94% were obtained for adrenaline and noradrenaline. The lower limit of detection for both the metanephrine and normetanephrine assays was 7 pg ml^−1^. Average intra-assay coefficients of variation were 7.95% for metanephrine and 6.45% for normetanephrine. Average recoveries of 94% and 95% were obtained for metanephrine and normetanephrine.

### Metabolic domains

On completion of the exercise tests metabolic and physiological data were grouped into domains to allow comparisons between participants relative to workload and physiological responses, namely (i) baseline (REST), (ii) two hours post ingestion at rest (POST-ING), (iii) highest lipid oxidation rate during exercise (FAT_peak_), (iv) lactate threshold, i.e. the value of LT estimated using simple linear regression by fitting to a model and identifying the workload LT, corresponding to the model with minimum Mean Squared Error (LT; calculated using Lactate-E software [34] and (v) peak rate of oxygen consumption (*V*O_2peak_).

### Calculations

From the recorded variables of *V*O_2_ and VCO_2_, fat and carbohydrate oxidation rates (g.min^−1^) were calculated using the stoichiometric equations at rest [32] and during exercise [35] under the assumption that protein utilisation was negligible.

### Data analysis

The data were analysed using the Statistical Package for the Social Sciences software (Version 26, SPSS, Inc). Data were reported as means ± SD with *P* ≤ 0.05 accepted. All data were assessed for normality (Shapiro–Wilk’s test) and subsequently analysed using two-way repeated measures ANOVAs (condition × time) with post hoc dependant *t* tests conducted with Bonferroni corrections where appropriate. Paired samples *t* test was used to compare performance parameters. This pilot study could not generate a priori power on primary endpoints due to lack of published information. However, in our pilot, post-priori data analysis on the primary catecholamine of interest (peak adrenaline concentration difference between EGCG and PLAC) revealed a statistical power of 99% in eight participants at an alpha error level (two-sided significance) of 5%.

## Results

### Cardio-respiratory changes and ratings of perceived exertion

Cardio-respiratory changes and rating of perceived exertion under both PLAC and EGCG at rest and during exercise are reported in Table 1. Although there was a clear effect of exercise intensity on ventilation, oxygen consumed, carbon dioxide produced and respiratory exchange ratio, heart rate and RPE there was no effect of supplementation on any of these cardio-respiratory variables (NS). Fat_peak_ occurred at a similar percentage of *V*O_2peak_ in both trials (PLAC 37.4 ± 4.8 vs. EGCG 37.4 ± 4.0%, NS). LT occurred at a similar percentage of *V*O_2peak_ (PLAC 79.5 ± 8.3 vs. EGCG 79.0 ± 9.9%, NS).

**Table 1:** Cardiorespiratory markers and rating of perceived exertion scores at rest and during exercise during the placebo and EGCG trials (*n* = 8)

Variable	Condition	Rest	Fat_peak_	LT	*V*O_2peak_
*V*_E_ (L.min^−1^)	PLAC	11.8 ± 4	28.4 ± 5	85.0 ± 24	139.4 ± 22
	EGCG	11.1 ± 4	32.1 ± 4	88.3 ± 22	141.6 ± 28
*V*O_2_ (L.min^−1^)	PLAC	0.3 ± 0.1	1.3 ± 0.2	2.9 ± 0.4	3.6 ± 0.6
	EGCG	0.3 ± 0.1	1.3 ± 0.2	2.8 ± 0.3	3.4 ± 0.8
*V*CO_2_ (L.min^−1^)	PLAC	0.3 ± 0.1	1.0 ± 0.2	2.7 ± 0.8	4.0 ± 0.6
	EGCG	0.3 ± 0.1	1.2 ± 0.2	3.0 ± 0.4	4.0 ± 0.8
RER	PLAC	0.86 ± 0.09	0.78 ± 0.1	1.03 ± 0.1	1.12 ± 0.1
	EGCG	0.88 ± 0.07	0.85 ± 0.1	1.05 ± 0.1	1.13 ± 0.04
HR (bpm)	PLAC	66 ± 8	100 ± 9	172 ± 9	191 ± 9
	EGCG	67 ± 9	105 ± 6	172 ± 9	191 ± 8
RPE (6–20)	PLAC		7 ± 2	16 ± 1	19 ± 0.7
	EGCG		7 ± 1	15 ± 2	18 ± 0.83

Only between group differences indicated. Statistical significance over time not reported for ease of interpretation

Data reported as mean ± SD

### Energy expenditure, carbohydrate and lipid oxidation

**Fig. 1:**
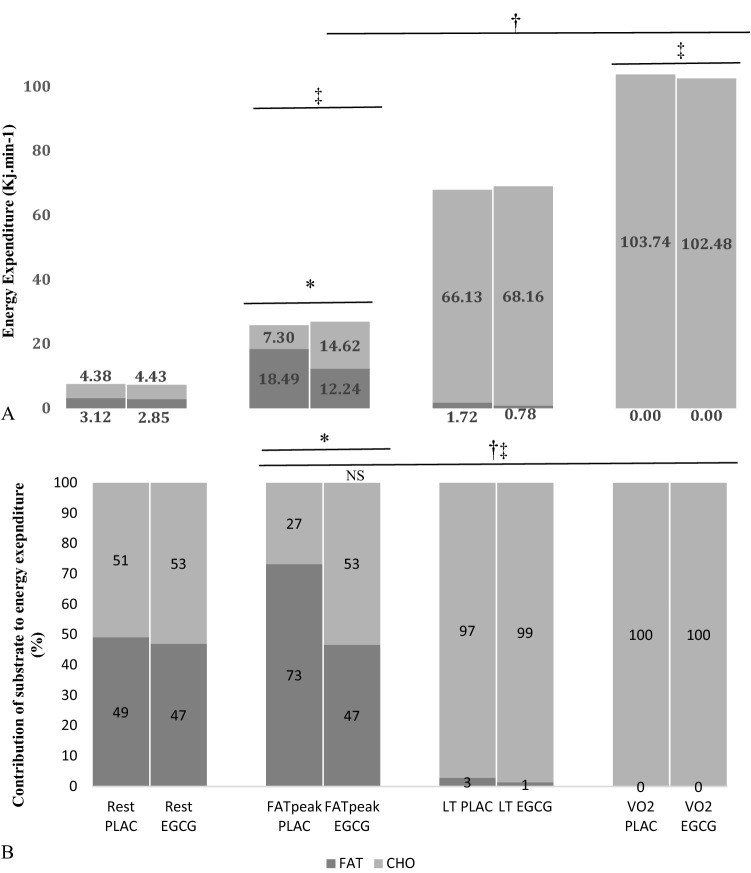
**A** Total energy expenditure and contribution from lipid (FAT) and carbohydrate (CHO) oxidation and **B** percentage contribution of lipid (FAT) and carbohydrate (CHO) oxidation to total energy expenditure; at rest, at highest lipid oxidation rate (FATpeak), point of lactate threshold (LT) and peak rate of oxygen consumption (*V*O_2peak_). *Indicates significant difference in energy derived from lipids and carbohydrate oxidation between conditions (*P* ≤ 0.05). †Indicates significant difference in energy derived from carbohydrate oxidation across time compared to rest (*P* ≤ 0.05), ‡indicates significant difference in energy derived from lipid oxidation across time compared to rest (*P* ≤ 0.05), unless annotated by non-significant time point (NS; **B**, Rest vs. FATpeak, EGCG treatment). Statistical analysis conducted using a 2-way repeated measures ANOVA and paired sample *T* Test

The resting and exercise-induced changes in energy expenditure, carbohydrate and lipid oxidation are located in Fig. 1. There was a 12–14-fold increase in oxidative energy expenditure during the graded cycle exercise test that was similar between conditions (NS). Interestingly, though an isoenergetic domain, rates of lipid oxidation at FAT_peak_ were reduced by 32% in the EGCG trial (EGCG 0.33 ± 0.14 vs. PLAC 0.49 ± 0.11 g.min^−1^, *P* < 0.05) with CHO utilisation correspondingly greater at this point (EGCG 0.87 ± 0.39 vs. PLAC 0.44 ± 0.34 g.min^−1^, *P* < 0.05).

### Blood lactate and glucose

**Fig. 2:**
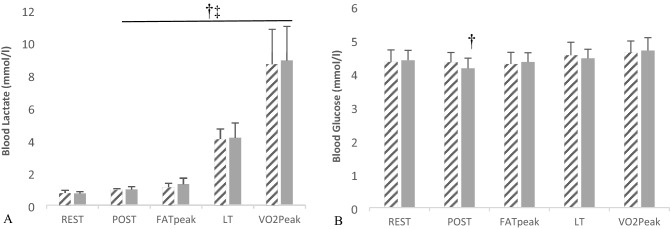
**A** Blood lactate & **B** blood glucose responses during the Placebo (diagonal line fill) and EGCG (grey) trials at rest, at post ingestion (POST) at highest lipid oxidation rate (FAT_peak_), point of lactate threshold (LT) and peak rate of oxygen consumption (*V*O_2peak_). †Indicates significant difference across time compared to rest in EGCG treatment only (*P* ≤ 0.05). ‡Indicates significant difference across time compared to rest in placebo treatment only (*P* ≤ 0.05). Data was analysed by repeated measures ANOVA with subsequent post hoc analysis, reported as mean ± SD

Blood lactate concentrations (Fig. 2) were influenced by time (*P* < 0.05) but not by trial (NS). At FAT_peak_ blood lactate rose by ~ 50% from rest within each condition and graded exercise produced a 12-fold increase in blood lactate under both PLAC and EGCG at *V*O_2peak_. Likewise, blood glucose concentrations were also influenced by time (*P* < 0.05), but did not differ between conditions (NS). Interestingly, under EGCG blood glucose post-ingestion was lower compared to resting values (EGCG Rest 4.39 ± 0.30 vs. Post-Ingestion 4.15 ± 0.30 mmol.l^−1^, *P* ≤ 0.05). There was a small rise in blood glucose concentration from rest to VO_2peak_ values in the PLAC trial (REST 4.34 ± 0.4 vs. *V*O_2peak_ 4.62 ± 0.3 mmol.l^−1^ *P* < 0.05) and also in the EGCG trial (REST 4.39 ± 0.3 vs. *V*O_2peak_ 4.68 ± 0.38 mmol.l^−1^ *P* < 0.05).

### Graded cycle test performance

There was no change in performance time noted between trials (EGCG 1370 ± 152 vs. PLAC 1377 ± 150 s, NS). All participants attained similar power outputs under both PLAC and EGCG conditions (EGCG 270 ± 32 vs. PLAC 266 ± 25 W, NS). Likewise, similar *V*O_2peak_ values were noted (PLAC 44.8 ± 4.7 vs. EGCG 43.0 ± 5.0 ml.kg.min^−1^, NS).

### Plasma catecholamine and catecholamine metabolites

**Fig. 3:**
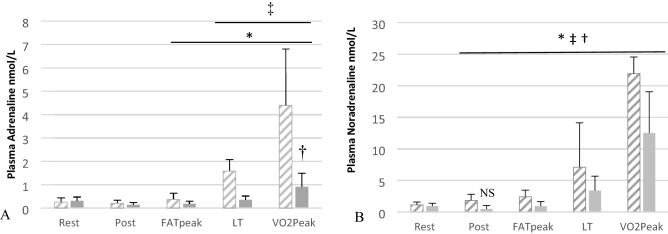
**A** Plasma adrenaline & **B** plasma noradrenaline responses during the placebo (diagonal line fill) and EGCG (grey) trials at rest, at post ingestion (POST-ING) at highest lipid oxidation rate (FAT_peak_), point of lactate threshold (LT) and peak rate of oxygen consumption (*V*O_2peak_). *Indicates significant difference between conditions at time point (*P* ≤ 0.05). †Indicates significant difference across time compared to rest in EGCG treatment only (*P* ≤ 0.05), unless annotated by non-significant time point (NS; **B**, Rest vs. Post). ‡Indicates significant difference across time compared to rest in placebo treatment only (*P* ≤ 0.05). Data was analysed by repeated measures ANOVA with subsequent post hoc analysis and reported as mean ± SD

Plasma AD and NA concentrations are reported in Fig. 3. Resting plasma AD concentrations were similar between conditions and were not altered following ingestion of either supplement. Post-ingestion NA concentrations were lower compared to resting values under both PLAC and EGCG (EGCG; Rest.vs. Post; 0.95 ± 0.44 vs. 0.48 ± 0.55 nmol.l^−1^, *P* < 0.05. PLAC; Rest.vs. Post; 1.15 ± 0.44 vs. 1.86 ± 0.94 nmol.l^−1^, *P* < 0.05). Compared to PLAC, EGCG plasma AD concentrations were statistically lower at FAT_peak_ (EGCG 0.18 ± 0.11 vs. PLAC 0.37 ± 0.27 nmol.l^−1^, *P* < 0.05), LT (EGCG 0.35 ± 0.16 vs. PLAC 1.59 ± 0.49 nmol.l^−1^, *P* < 0.001) and *V*O_2peak_ (EGCG 0.91 ± 0.58 vs. PLAC 4.39 ± 2.42 nmol.l^−1^, *P* < 0.001). NA concentrations under EGCG were significantly lower at FAT_peak_ (EGCG 0.93 ± 0.72 vs. PLAC 2.43 ± 1.04 nmol.l^−1^, *P* < 0.05), LT (EGCG 3.41 ± 2.27 vs. PLAC 7.12 ± 7.00 nmol.l^−1^, P < 0.01), and *V*O_2peak_ (EGCG 12.52 ± 6.53 vs. PLAC 21.90 ± 2.66 nmol.l^−1^, *P* < 0.05).

**Fig. 4:**
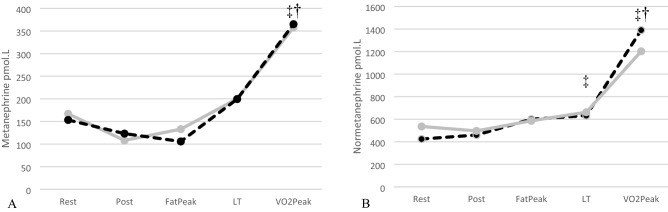
**A** Metanephrine and **B** normetanephrine responses during the Placebo (black dashed) and EGCG (grey solid) trials at rest, at post-ingestion (POST) at highest lipid oxidation rate (FAT_peak_), point of lactate threshold (LT) and peak rate of oxygen consumption (*V*O_2peak_). †Indicates significant difference across time compared to rest in EGCG treatment only (*P* ≤ 0.05). ‡Indicates significant difference across time compared to rest in placebo treatment only (*P* ≤ 0.05). Data were analysed by repeated measures ANOVA with subsequent post hoc analysis and reported as mean values

Resting MET and NORMET concentrations were similar and did not change after 2 h of ingestion of either supplement (NS). Resting MET concentrations increased similarly to 321 ± 235 pmol.l^−1^ (NS) at *V*O_2peak_ (Fig. 4) and in the EGCG trial to 358 ± 215 pmol.l^−1^ (*P* < 0.05). NORMET concentrations rose progressively with exercise in both trials reaching similar peak values (EGCG 1203 ± 500 vs. PLAC 1390 ± 598 pmol.l^−1^, NS).

## Discussion

This study explored the impact of acute ingestion of the polyphenol epigallocatechin gallate (EGCG) on catecholamine, catecholamine metanephrine, systemic metabolic and cardio-respiratory variables during continuous incremental cycle exercise. To the authors’ knowledge, this study is the first to demonstrate that EGCG supplementation resulted in lowered circulating catecholamine concentrations. In addition, EGCG altered oxidative energy provision from lipid and carbohydrate at low exercise intensities.

Increased physical exercise intensity elevated heart rate, ventilation, O_2_ consumption, CO_2_ production and ratings of perceived exertion. Research studies that administered green tea extract to human participants found no change [18, 36] or lower heart rates [23] in response to exercise when compared to a placebo. Our results agree with the findings [18, 36], where exercise-induced increases in heart rate were similar between control and EGCG conditions. In contrast, the differing findings [23] might be explained by the polyphenol mixture administered by the researchers compared to only EGCG (94% pure) in our study. It might be suggested that the lower circulating catecholamine concentrations under EGCG would reduce HR, however, that other hormonal, humoral or electrical stimulants of sino-atrial node pacing influence heart rate suggest a greater contribution of these other factors to heart rate regulation during exercise.

Using principles of stoichiometry to determine the lipid and carbohydrate proportions of energy utilisation during exercise at relatively similar metabolic domains, this study revealed a 32% decreased oxidation rate of lipids following EGCG supplementation at FAT_peak_ compared to placebo. Increased *V*_E_ and raised *V*CO_2_ rates under EGCG help explain this finding and led to a greater respiratory exchange ratio (RER) value (PLAC 0.78 ± 0.05 vs. EGCG 0.85 ± 0.07) that is indicative of a lower rate of lipid oxidation (Fig. 1) and a compensatory increase in carbohydrate oxidation rate under EGCG at FAT_peak._ This is a novel finding and is contrary to previous studies where EGCG-containing supplements increased lipid oxidation during exercise [16, 17] or did not influence substrate utilisation [18, 20, 22, 23]. It is difficult to fully reconcile our data with those of other researchers but suggest some of the differences may be due to the different exercise protocols, dosages, timings of supplement administration, percentage EGCG in GTE and/or caffeine-containing GTE.

The lipids (non-esterified fatty acids) oxidised during exercise are derived from intramuscular depots or adipose tissue. However, during exercise above the lactate threshold, circulating FFA amounts cannot meet the tissue oxidative needs and intramuscular triglyceride stores are used more [37]. In both adipose and skeletal muscle tissues, the enzyme hormone sensitive lipase (HSL) stimulates triglyceride breakdown and consequent liberation of free fatty acids for oxidation. During exercise, intramuscular HSL activity is stimulated by muscle contraction [38] and adrenaline [25, 39] with the effects of both factors being additive [40]. Given the exercise characteristics were well controlled in our study (cadence 60 rpm, 30 W progressive power increase every 3 min), the decreased rate of lipid oxidation at FAT_peak_ which occurred at a similar relative exercise intensity (PLAC 37.4 ± 4.8 vs. EGCG 37.4 ± 4.0%*V*O_2peak_) might be due to a lesser stimulation of hormone sensitive lipase on intramuscular triglyceride utilisation via a decreased sympatho-adrenal response.

Many studies researching green tea extract or EGCG have explored oxidative energy provision with scant attention paid to non-oxidative pathways. We measured resting and exercising blood lactate concentration as an indicator of non-oxidative energy metabolism. As graded exercise was mostly performed in an oxidative isoenergetic domain, the noted difference in RER between PLAC and EGCG might indicate an increase in CHO utilisation. Increased CHO flux through non-oxidative glycolysis preceding oxidation might result in elevated pyruvate and lactate formation, with an increase in both these markers [28], following green tea extract supplementation. That there were no between-group differences in blood lactate is interesting especially despite the dramatically lowered circulating catecholamine values and suggests a lesser role of catecholamines in stimulating production of muscle glycogenolysis compared to insulin suppression and increased circulating glucagon [41]. However, given the absolute lactate concentrations in the bloodstream at the point of highest lipid oxidation (FAT_peak_) were low (~ 1 mM) our data must be interpreted with some caution. The lack of change in blood lactate contradicts previous work by Hodgson et al. [28], that in response to a 60-min cycle at 56% *V*O_2max_ there was an increase in blood lactate concentrations following GTE supplementation; however, they noted no change in catecholamine concentrations, contrary to this study. Blood glucose levels after ingestion of EGCG were lower compared to rest (~ 0.2 mmol.l^−1^). This decrease may be due to an insulin sensitising effect of EGCG, with literature demonstrating a 13% increased insulin sensitivity in the fed state following acute EGCG supplementation [16]. Alternatively, plasma catecholamines stimulate hepatic β-adrenoreceptors and increase glycogenolysis with increased glucose release to the bloodstream. That resting plasma NA was lower under EGCG might help explain the lower blood glucose concentration at this time point.

There was no change in performance time or power output after EGCG supplementation. This is a similar finding to previous research [18] and provides little evidence of ergogenic potential of EGCG in exercise performance. Given the complex regulatory, often compensatory processes involved in the provision of oxygen and fuel during physical exercise a small alteration in lipid use alongside reductions in sympatho-adrenal activity did not materialise in any alteration in the perception of exertion or functional capacity.

Green tea leaves contain components like caffeine that can alter circulating catecholamine concentrations in some [4, 42] but not all [3, 5, 8, 28] studies so is a confounding variable. Thus, in our study we explored the influence of high purity (94%), low caffeine (< 0.1%) EGCG extract (the highest concentration polyphenol in green tea) on resting and exercising circulating catecholamines and catecholamine metabolites (that directly involve the activity of catechol-O-methyl transferase). Consumption of EGCG via capsule form generates an average peak plasma concentration (*C*_max_) 2.5–2.8 h post-consumption [43], this corresponds to circulating catecholamine AD concentrations being greatly lowered following acute EGCG supplementation at optimum bioavailability when compared to PLAC, with plasma AD concentrations at (i) FAT_peak_ reduced by 53%, (ii) LT being reduced by 78%, and (iii) *V*O_2peak_ being reduced by 79%. Although it has long been recognised that flavanoids inhibit COMT activity [29], only more recently has EGCG been shown to inhibit human hepatic cytosolic COMT activity in vitro [27, 44]. Inhibition of COMT could allow for sustained (if not elevated) plasma catecholamine concentrations and potentially reduce production of downstream catecholamine metabolites (metanephrine and normetanephrine). The results of our study throw into question this mechanism. As stated above, plasma catecholamines were greatly reduced after EGCG supplementation and we found no differences in the resting or exercise-induced increases in plasma metanephrine or normetanephrine compared to the placebo trial suggestive of similar COMT activity in each trial. Thus, the mechanism(s) by which plasma circulating catecholamines are lower during exercise following EGCG supplementation are unclear. EGCG (as opposed to green tea extract) has been shown to have no influence α-adrenergic stimulation of rat vascular and aortic tissue in vitro [45]. Yet catecholamine secretion from perfused rat adrenal medulla is reduced due to the influence of polyphenols (that also contained epicatechin) via interruption of sodium and calcium interchange in chromaffin tissue and/or inducing nitric oxide release in vascular tissue [46]. Adding to the complexity of responses, administration of caffeine to rats with or without EGCG reduced the increase in catecholamines and vascular responses associated with caffeine administration alone [47]. As an alternative suggestion to explain our data, EGCG has been shown to inhibit DOPA decarboxylase, slowing conversion of l-DOPA into dopamine; the upstream product of NA and AD within adrenal medulla chromaffin cells and sympathetic neuron [48]. However, further investigation is warranted to detail the exact in vivo mechanistic pathways involved during exercise in man.

In conclusion, this study explored the impact of acute ingestion of the polyphenol epigallocatechin gallate (EGCG) on catecholamine, catecholamine metanephrines, systemic metabolic and cardio-vascular variables across a range of exercise intensities during graded cycle exercise in man. Circulating catecholamine concentrations and peak lipid oxidation were lower in response to a graded exercise test following acute EGCG supplementation.

Study limitations: Blood samples collected at the end of each stage were able to be analysed for catecholamines and catecholamine metabolites; however, we established five metabolic time points which were examined to remove potential bias and mitigated this limitation, all samples were however analysed for plasma blood lactate and plasma blood glucose. Second, the lack of a post ingestion respiration sample, in any future study it would be beneficial to add this sample point to allow a more complete picture of any subtle changes which may have occurred during the rest period.

## Data Availability

The data that support the findings of this study are not openly available due to reasons of sensitivity i.e. human data and are available from the corresponding author upon reasonable request from a controlled access repository.
